# Loss of CD24 in Mice Leads to Metabolic Dysfunctions and a Reduction in White Adipocyte Tissue

**DOI:** 10.1371/journal.pone.0141966

**Published:** 2015-11-04

**Authors:** Nicholas A. Fairbridge, Thomas M. Southall, D. Craig Ayre, Yumiko Komatsu, Paula I. Raquet, Robert J. Brown, Edward Randell, Christopher S. Kovacs, Sherri L. Christian

**Affiliations:** 1 Department of Biochemistry, Faculty of Science, Memorial University of Newfoundland, St. John’s, Newfoundland, Canada; 2 Division of BioMedical Sciences, Faculty of Medicine, Memorial University of Newfoundland, St. John’s, Newfoundland, Canada; 3 Department of Laboratory Medicine, Faculty of Medicine, Memorial University of Newfoundland, St. John’s, Newfoundland, Canada; 4 Division of Medicine-Endocrinology, Faculty of Medicine, Memorial University of Newfoundland, St. John’s, Newfoundland, Canada; College of Tropical Agriculture and Human Resources, University of Hawaii, UNITED STATES

## Abstract

CD24 is a glycophosphatidylinositol (GPI)-linked cell surface receptor that is involved in regulating the survival or differentiation of several different cell types. CD24 has been used to identify pre-adipocytes that are able to reconstitute white adipose tissue (WAT) *in vivo*. Moreover, we recently found that the dynamic upregulation of CD24 *in vitro* during early phases of adipogenesis is necessary for mature adipocyte development. To determine the role of CD24 in adipocyte development *in vivo*, we evaluated the development of the inguinal and interscapular subcutaneous WAT and the epididymal visceral WAT in mice with a homozygous deletion of CD24 (CD24KO). We observed a significant decrease in WAT mass of 40% to 74% in WAT mass from both visceral and subcutaneous depots in male mice, with no significant effect in female mice, compared to wild-type (WT) sex- and age-matched controls. We also found that CD24KO mice had increased fasting glucose and free fatty acids, decreased fasting insulin, and plasma leptin. No major differences were observed in the sensitivity to insulin or glucose, or in circulating triglycerides, total cholesterol, HDL-cholesterol, or LDL-cholesterol levels between WT and CD24KO mice. Challenging the CD24KO mice with either high sucrose (35%) or high fat (45%) diets that promote increased adiposity, increased WAT mass and fasting insulin, adiponectin and leptin levels, as well as reduced the sensitivity to insulin and glucose, to the levels of WT mice on the same diets. The CD24-mediated reduction in fat pad size was due to a reduction in adipocyte cell size in all depots with no significant reduction pre-adipocyte or adipocyte cell number. Thus, we have clearly demonstrated that the global absence of CD24 affects adipocyte cell size *in vivo* in a sex- and diet-dependent manner, as well as causing metabolic disturbances in glucose homeostasis and free fatty acid levels.

## Introduction

Obesity, caused by excessive caloric intake and exacerbated by both environmental and genetic factors [1], is an increase in the amount of white adipose tissue (WAT) to pathological levels. In contrast, lipodystrophy is either a generalized or localized loss of WAT induced by genetic or environmental factors [2]. In both situations the increased circulation of lipotoxic free fatty acids (FFA) and the reduced ability to regulate blood glucose levels promotes co-morbidities such as diabetes, stroke, and cardiovascular disease.

WAT stores excess energy in the form of triglycerides (TG), as well as being an endocrine organ that secretes appetite regulating hormones such as adiponectin and leptin [3]. WAT is located in multiple depots, including subcutaneous depots beneath the skin and visceral depots surrounding internal organs. Mature adipocytes in WAT are composed of a unilocular lipid droplet (LD), and a thin ring of cytoplasm with a flattened nucleus [4].

Adipocytes can increase WAT size through a combination of increased cell size (hypertrophy) and increased cell number (hyperplasia) [3]. During hypertrophy, there is an increase in the amount of neutral lipids, mainly TG and cholesteryl esters, stored in the WAT LDs that markedly increases overall cell size [4]. Hypertrophy can occur throughout life in response to excess energy intake [5]. Hyperplasia is a process that normally occurs during early development as well as in childhood and adolescence in response to excess caloric intake [5]. Given that the number of adipocytes in adults remains stable with approximately 10% of the adipocyte population being turned over each year [5], the primary mechanism for the increase in WAT size in adulthood is hypertrophy. Understanding the molecular mechanisms underlying the regulation of hyperplasia and hypertrophy is necessary in order to develop therapies that can target obesity or lipodystrophy.

During early development and adipocyte turnover in adults [5], multi-potent mesenchymal stem cells will differentiate into mature adipocytes [6]. Cells committed to the adipocyte lineage arise from the perivascular region within WAT [7]. Differentiation of adipose-derived stem cells or pre-adipocytes into mature lipid-laden adipocytes is a multi-stage process. Pre-adipocytes first undergo clonal expansion and then exit the cell cycle prior to induction of transcriptional cascades that activate ‘master regulators’ of adipogenesis, CCAAT enhancer binding protein α (C/EBP-α) and peroxisome proliferator-activated receptor γ (PPAR-γ) [8–11]. Different WAT depots show varying degrees of differentiation potential [3] with subcutaneous WAT found to contain a greater number of pre-adipocytes than epididymal WAT [12].

In response to fasting, lipolysis in WAT causes the release of FFA for energy production via fatty acid oxidation. In contrast, the action of insulin on WAT promotes TG storage via *de novo* FA synthesis, as well as promoting uptake of dietary TG from the circulation [13]. Therefore, TG in the WAT are a mix of dietary and synthesized lipid. In contrast, the action of leptin restricts *de novo* lipogenesis while allowing dietary TG uptake [13]. Furthermore, large adipocytes, which contain more TG in large LD, are associated with insulin resistance while small adipocytes are more responsive to insulin [14]. Thus, both the number and size of adipocytes influences the insulin-sensitivity of WAT and the ability to store TG.

Lipodystrophies and fat storage disorders in humans can occur in response to environmental or genetic factors that affect WAT in a generalized, or localized manner [15]. Genetic forms of lipodystrophy are rare and have been attributed to mutations in a number of genes that regulate LD synthesis, LD metabolism, adipocyte apoptosis, and adipogenesis. For example, mutations in *AGPAT2*, *BSCL2*, or *CAV1* cause congenital generalized lipodystrophy (CGL) due to deficiencies in LD synthesis and/or adipogenesis [2]. Mutations in the major transcriptional regulator of adipogenesis, PPAR-γ, cause familial partial lipodystrophy (FPLD) along with severe insulin resistance and hypertension [15]. Treatment of HIV patients with first generation protease inhibitors can cause partial lipodystrophy, primarily affecting subcutaneous WAT due to direct effects on adipocytes [15]. Causal mutations in up to 95% of patients with CGL [2] have been identified suggesting that other genetic causes remain to be identified. Many cases of FPLD lipodystrophy have no known causal mutations [15] and mild cases of fat storage disorders are likely to remain undiagnosed in the absence of additional co-morbidities.

Previously, a subpopulation of adipocyte progenitor cells was identified with the expression of the cell surface molecule CD24 being critically important for reconstitution of WAT function *in vivo* [16]. CD24 is a heavily glycosylated glycosylphosphatidylinositol (GPI)-linked cell surface receptor found on a variety of cell types including cancer cells, precursor B lymphocytes, neurons, epithelial cells, macrophages and pre-adipocytes [17]. CD24 can also regulate cell survival and differentiation in a cell-type dependent manner [17].

Recently, we have demonstrated *in vitro* that both CD24 mRNA and surface protein is dramatically increased *in vitro* in primary cells and in the 3T3-L1 pre-adipocyte cell line early during adipogenesis [18]. Blocking the increase in CD24 significantly inhibited subsequent adipogenic gene expression and lipid accumulation [18]. Furthermore, we observed a clear loss in CD24 expression as the pre-adipocytes commit to the adipocyte lineage *in vitro* [18], which is consistent with the loss of CD24 expression observed in mature adipocytes *in vivo* [19] and the rapid gain and loss of CD24-positive adipocyte progenitor cells in response to high fat diet *in vivo* [20]. However, the role that CD24 plays in the regulation of adipogenesis during WAT hyperplasia or hypertrophy growth and development *in vivo* is not known. We analyzed the size and structure of WAT in the CD24 knock-out (CD24KO) mouse on the diet-induced-obesity prone C57BL/6 background (C57BL/6N *Cd24* ^*atm1Pjln*^ *)* when fed a standard chow diet (chow), high-sucrose/low fat diet (HSD), or high-fat/low sucrose diet (HFD). We also analyzed the effect of aging and/or diet on key markers of adipocyte function in the CD24KO mice.

## Materials and Methods

### Animals and diets

All animal use was approved by the Institutional Animal Use and Care Committee at Memorial University of Newfoundland (protocol 14-05-SC). Previously described C57BL/6N *Cd24* ^*atm1Pjln*^ homozygous mice [21,22], were a gift from Dr. Yang Liu (Center for Cancer & Immunology Research, Children’s National Medical Center, Washington, DC). C57BL/6N mice were purchased from Charles River (Saint Constant, QC, Canada). Mice were maintained on standard ProlabRMH 3000 rodent chow (chow, 4% sucrose, 60% carbohydrate, 14% fat [by kcal], Lab Diet, St, Louis, MO) until 4 weeks of age, at which point the diet was either switched to HSD (35% sucrose, 70% carbohydrate, 10% fat [by kcal] D12450B, Research Diets, New Brunswick, NJ), or HFD (17% sucrose, 35% carbohydrate, 45% fat [by kcal], D12451, Research Diets), or continued on chow, *ad libitum*. Mice were housed 2–3 per cage in microisolator cages, on a 12 h:12 h dark:light light cycle, with free access to water, and randomly assigned to diets. Mice were assessed bi-weekly for overall health and welfare. Two WT mice fed chow diet showed obvious signs of distress and were removed from the study. All assessments were done on matched sets of WT and CD24KO mice with samples taken alternately from each genotype.

### Dual-energy X-ray absorptiometry

Male and female mice at 5, 9 and 12 weeks of age were anesthetized with isoflurane followed by intraperitoneal (i.p.) injection of 0.1 mg/g ketamine and 0.2 mg/g xylazine to body weight. Animals were positioned prone with limbs and tail adjusted to standardized positions and scanned on a PIXImus bone densitometer (LUNAR, Madison, WI). The skull was excluded from the scan.

### Physiological assessments

Glucose tolerance tests (GTT) were conducted following a 6 h fast [23], and animals were injected i.p. with 2 g/kg D-glucose in sterile PBS. Forty-eight hours later, insulin tolerance tests (ITT) were conducted following a 4 h fast [23], and animals injected i.p. with 1 U/kg of insulin from bovine pancreas (Sigma-Aldrich, ON, Canada) in sterile PBS. For GTT and ITT, blood glucose from the tail was determined at 15, 30, 60 and 120 min post-injection, using a Freestyle Lite glucose meter (Abbott Diabetes Care, ON, Canada), which measures blood glucose concentrations up to 28 mM. Measurements above the detection limit were set to 28 mM for statistical analyses. Overall glucose load was determined by calculating total area under the curve, while the glucose response was calculated as area from the initial fasting blood glucose level.

Animals were euthanized at either 5, 9, or 15 weeks of age following a 6 h fast. Blood plasma samples were collected by cardiac puncture into EDTA (50 μM) under isoflurane anesthesia, followed by cervical dislocation. Blood was centrifuged immediately following collection for 10 min at 2000 x g and stored at -20°C. Plasma total cholesterol (TC), high density lipoprotein cholesterol (HDL-C), TG, and pancreatic lipase levels were determined using an Architect c16000 clinical chemistry analyzer (Abbott Diagnostics, Lake Forest, IL, USA) following the manufacturer’s instructions. LDL-cholesterol (LDL-C) was derived following the method by Friedewald *et al*. [24]. Plasma insulin levels were determined using the Rat/Mouse Insulin ELISA (Millipore, Etobicoke, ON, Canada) following the manufacturer’s instructions. Plasma adiponectin and leptin levels were determined using DuoSet kits from R&D Systems, Inc. (Minneapolis, MN) following the manufacturer’s instructions. Total FFA were quantified using the NEFA-HR(2) kit (Wako Diagnostics, Richmond, VA, USA) from 4 μl plasma, with 0–4 nmol oleic acid used to generate a standard curve.

Homeostatic model assessment (HOMA) for insulin resistance (IR) was calculated as mM glucose x ng/mL insulin/22.5 and HOMA-β for β-cell function as (20 x pg/mL insulin)/(mM glucose -3.5) [25].

### Morphological assessments

Body weight, body length, liver, inguinal, interscapular, and visceral epididymal fat pad weight were determined at 9 weeks of age. WAT and pancreas were fixed in 10% neutral buffered formalin, followed by staining of paraffin-embedded tissue sections with Heamatoxylin and Eosin (H&E) following standard procedures by the Medical Education and Laboratory Support Services (Memorial University). Images were acquired using an Axio Imager.A1 microscope with an AxioCam HR Rev3 using AxioVision software v 4.8.2.0 (Carl Zeiss Microscopy). WAT cell area was calculated using CellProfiler image analysis software [26]. Cell number was estimated from the mass of the WAT depots following the method of Jo *et al*. [27] except that volume was calculated from the measured area as described previously [28]. Pancreatic islet size was determined using ImageJ software [29].

### Statistical analysis

Statistical analysis was performed in R v.3 [30]. Differences in cell size distributions were tested using the Kolmogrov-Smirnov test. Pairwise comparisons of non-parametric data were performed using the Wilcox rank sum test. Pairwise comparisons of parametric data was analyzed by Student’s t-test. All other analyses were performed using repeated measures ANOVA or linear mixed model [31], as indicated. Differences were considered statistically significant at P<0.05.

## Results

### Male, but not female, CD24KO mice show a reduction in fat mass

**Fig 1:**
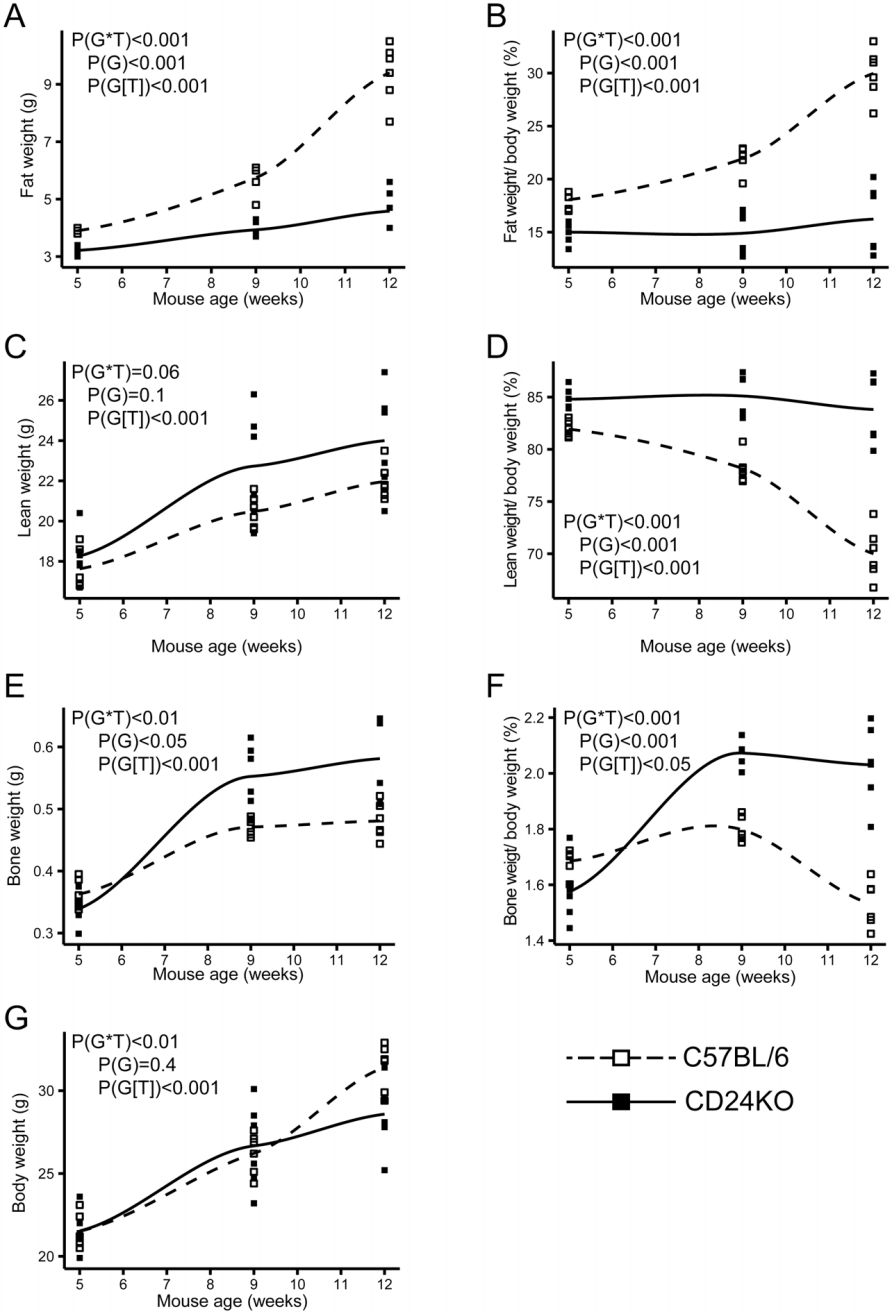
CD24KO male mice have reduced fat and increased lean and bone weight compared to wild-type male mice. **A.** Total fat weight, **B.** percent fat weight, **C.** total lean weight, **D.** percent lean weight, **E.** bone weight, **F.** percent bone weight, and **G.** estimated total body weight from dual X-ray absorptiometry (DEXA) scans of male wild-type C57BL/6 and CD24KO mice at 5, 9, and 12 weeks of age on standard show diet. Trend lines display the Loess conditional means and squares represent individual animals. Statistical significance determined by repeated measures ANOVA, n = 6, interaction effects between genotype (G) and time (T) are indicated as P(G*T), main effects of genotype are shown as P(G) and the effect of time within each genotype is shown as P(G[T]).

To determine the contribution of CD24 to fat cell development *in vivo*, we first analyzed the overall gain of fat, lean tissue, and bone mass using dual x-ray absorptiometry (DEXA) in CD24KO mice, from 5 weeks to 12 weeks of age on chow diet. We found that male CD24KO had significantly less overall fat and percentage body fat with a difference that increased over time when compared to age and sex-matched wild-type (WT) control mice (Fig 1A and 1B). There was a concomitant significant increase in the amount of lean and bone mass in the same animals (Fig 1C–1F). In the estimate of total body weight derived from the DEXA scans, we found that the mice had similar weights at 5 and 9 weeks of age, which diverged by 12 weeks with CD24KO mice weighing 91±6% of their WT counterparts at that age (Fig 1G). These data suggest that male CD24KO mice are deficient in the overall accumulation of fat that occurs during aging but that there is general compensation in lean and bone mass, which tends to wane by 12 weeks of age.

In female CD24KO mice, we found that there was no clear decrease in total fat mass over time (S1A Fig) but there was less percentage fat in CD24KO female mice (S1B Fig). Lean mass and percentage lean mass showed the opposite trend with female CD24KO mice showing an overall significant increase in lean mass (S1C and S1D Fig). Therefore, the apparent reduction in the percentage of fat mass was due to the increase in lean tissue. We observed no effect of CD24KO on bone mass in female mice (S1E and S1F Fig), or any effect of CD24KO on overall mass (S1G Fig). Therefore, female CD24KO mice are spared the CD24-mediated reduction in WAT.

**Fig 2:**
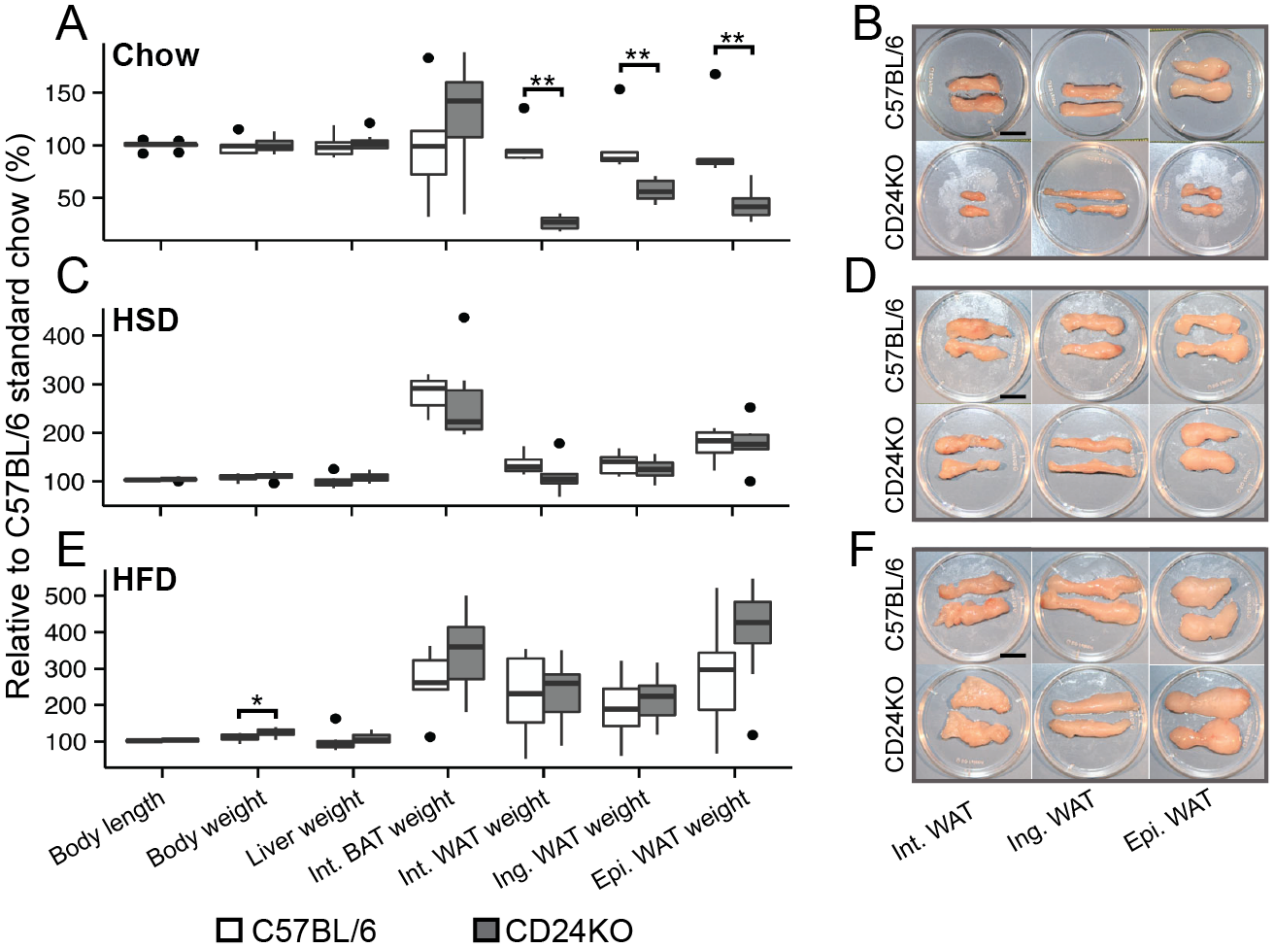
Impaired fat mass accumulation in CD24KO affects all WAT depots and is rescued by adiposity-promoting diets. Male CD24KO and wild-type mice were fed **A-B**. standard chow diet (chow), **C-D.** high sucrose diet (HSD), or **E-F.** high fat diet (HFD) for 5 weeks starting at 4 weeks of age. **A, C, E.** Relative body length, total weight, and weights of liver, interscapular (Int.) brown adipose tissue (BAT), Int. white adipose tissue (WAT), inguinal (Ing.) WAT, epididymal (Epi.) WAT are shown. Data are relative to the mean of the WT chow fed group and are shown as box-and-whisker plots. Statistical significance was determined by Wilcoxon rank sum, n = 5–9, *P<0.05, **P<0.01. **B, D, F.** Representative images of whole WAT from Int, Ing, and Epi WAT depots from 9-week-old CD24KO and WT male mice. Scale bar = 1 cm

We confirmed the data from the DEXA scans by physical assessment of fat pads from 9-week-old male mice since there was a reduction in fat pad weight with no overall loss in body weight at this age. We found that there was a substantial and significant decrease in the weight of two subcutaneous WAT depots (inguinal and interscapular) as well as the major visceral WAT (epididymal) in the CD24KO mice (Fig 2A and 2B). However, no significant difference was observed for interscapular BAT weight (Fig 2A and S2A Fig). We also found no difference in body length, body weight, or liver weight (Fig 2A). There were also no differences in fat accumulation in the liver (S2C and S2D Fig). Therefore, the CD24-mediated reduction in fat mass affects both subcutaneous and visceral WAT in a similar manner while sparing BAT, liver and overall body size.

Previous studies from our lab and others [16,18] have shown that the presence of CD24 can modulate adipogenesis in a context-dependent manner. Therefore, we next asked if feeding diets that promote adiposity to CD24KO mice could rescue the reduction in fat pad mass. The HSD induces lipogenic gene expression in the liver [32], which increases the transport of TG via very low-density lipoproteins (VLDL) to the WAT and leads to increased WAT size [33]. The HFD increases transport of dietary lipids via chylomicrons from the intestine to the WAT and also increases WAT size [33]. We fed male mice one of these diets from 4 weeks to 9 weeks of age. As expected, after 5 weeks on either diet all three fat pads from the WT mice increased in weight (Fig 2C–2F). With both HSD and HFD, the fat pads from CD24KO mice increased to a size that was statistically similar those of the WT mice (Fig 2C–2F). Therefore, WAT from male CD24KO mice can respond to increased circulating TG from both VLDL and chylomicron sources. As expected, the accumulation of fat in the liver significantly increased with both HSD and HFD; however, there was no significant difference between the WT and CD24KO mice (S2C and S2D Fig).

### CD24KO mice have diet-dependent alterations in glucose and lipid metabolism

In order to determine if the reduction in WAT mass experienced by male CD24KO mice resulted in alterations to WAT function, we analyzed markers of glucose metabolism, lipid metabolism, and select adipokines in plasma from CD24KO and WT mice at 5, 9, and 15 weeks of age (Table 1). We found a significant effect of genotype on fasting glucose, with significant increases in CD24KO mice at 5 weeks and 15 weeks of age. Fasting insulin levels were similarly decreased at 9 and 15 weeks of age with a significant interaction between genotype and age where the difference became more pronounced in older animals. HOMA-IR showed an overall effect of age but not genotype indicating that older mice became more insulin resistant but that the lack of CD24 did not significantly affect this progression. In contrast, HOMA-β levels were lower in CD24KO mice with a significant interaction between age and genotype, indicating that CD24KO mice tend to have lower β-cell function compared to WT animals.

**Table 1:** Indicators of glucose and lipid metabolism in fasted WT and CD24KO mice.

	5 weeks of age		9 weeks of age		15 weeks of age	
	C57BL/6	CD24KO	C57BL/6	CD24KO	C57BL/6^‡^	CD24KO
**Glucose (mM)** ^†^ ^a^	6.7±0.4	9.6±0.5*	7.9±0.9	9.2±0.8	5.9±1.1	9.9±0.8*
**Insulin (pg/mL)** ^†^ ^a^ ^b^ ^c^	778±65	724±49	483±69	337±49*	1553±180	756±81*
**HOMA-IR** ^†^ ^b^	0.23±0.02	0.31±0.03*	0.19±0.05	0.14±0.03	0.42±0.09	0.34±0.07
**HOMA-β (%)** ^†^ ^a^ ^b^ ^c^	5.66±0.72	2.63±0.31*	1.71±0.08	1.32±0.23	17.13±3.46	2.58±0.62*
**Adiponectin (ng/mL)** ^a^ ^b^ ^c^	3501±223	2955±146*	6764±426	4728±310*	6624±294	4764±445*
**Leptin (pg/mL)** ^§^ ^a^ ^b^ ^c^	n.d.	n.d.	93.6±27.7	n.d*	581.6±94.6	83.3±13.2*
**Free Fatty Acids (μM)** ^a^	117±1	145±1*	119±1	147±2*	122±3	145±1*
**TC (mM)**	n.a.	n.a.	1.59±0.06	1.56±0.16	1.82±0.12	1.54±0.06
**HDL-C (mM)**	n.a.	n.a.	0.88±0.05	0.85±0.09	0.91±0.07	0.72±0.03*
**LDL-C (mM)**	n.a.	n.a.	0.46±0.03	0.51±0.06	0.53±0.02	0.55±0.02
**TG (mM)** ^b^	n.a.	n.a.	0.54±0.05	0.49±0.03	0.83±0.07	0.62±0.07
**Pancreatic lipase (U/L)**	n.a.	n.a.	26±2	32±6	28±6	30±4
**Pancreatic Islet (μm** ^**2**^ **)** ^†^	n.a.	n.a.	8.58 ± 0.7	7.03 ± 0.60	n.a.	n.a.

mean ±sem, n = 6,

^‡^n = 3,

^†^n = 6 to 12.

^a^Main effect of genotype at P<0.05.

^b^Main effect of age at P<0.05.

^c^Interaction between genotype and age at P<0.05 by linear mixed model analysis.

*P<0.05 *a priori* analysis by Wilcoxon rank sum test between WT and CD24KO within each age.

^§^detection threshold of 62.5 pg/mL, n.a. = not analyzed, n.d. = not detected.

With respect to fat metabolism, we found that there were significantly increased levels of circulating FFA at all ages in the CD24KO mice. Plasma HDL-C was decreased at 15 weeks, however TC, LDL-C, and TG levels were unaffected at any age examined. Therefore, overall lipid transport appears to be maintained in the absence of CD24. Both leptin and adiponectin levels were significantly reduced in CD24KO mice in an age-dependent manner, most likely a direct reflection of the overall WAT mass. Pancreatic lipase activity, an estimate of pancreas damage, was slightly elevated in CD24KO mice but not to a significant level. We observed no significant effect of CD24KO in pancreatic islet size in 9-week-old mice (Table 1).

Since the adiposity-promoting diets rescued the CD24-dependent reduction in fat pad mass in 9-week-old animals, we analyzed the key glucose and fat metabolism markers in 9-week-old mice that had been on a HSD or HFD for 5 weeks (Table 2). Fasting glucose and FFA remained elevated in CD24KO mice on both diets, while insulin levels in CD24KO mice increased to levels similar to those in WT mice on the same diets. Adiponectin levels remained lower in CD24KO mice on HSD but not HFD, while leptin increased to WT levels in CD24KO mice on HSD and significantly above WT levels in CD24KO mice on HFD. The relative adipokine levels reflected the relative amounts of WAT in CD24KO mice compared to WT mice on the same diets. Pancreatic lipase levels became statistically different with HFD, but they remained within clinically acceptable levels of within three times the normal level [34]. HSD induced an increase in pancreatic islet size irrespective of genotype but neither genotype showed altered islet size when fed HFD (Table 2).

**Table 2:** Indicators of glucose and lipid metabolism in fasted WAT and CD24KO mice at 9 weeks of age after being fed high caloric diets for 5 weeks.

	High sucrose diet		High fat diet	
	C57BL/6	CD24KO	C57BL/6	CD24KO
**Glucose (mM)** ^†^ ^a^	6.8±0.7	8.8±0.7*	7.5±0.4	9.2±0.6*
**Insulin (pg/mL)** ^b^	859±222	728±105	965±133	941±98
**HOMA-IR** ^b^	0.24±0.06	0.29±0.06	0.33±0.06	0.38±0.6
**HOMA-β (%)** ^a^ ^b^	7.01±2.43	2.95±0.40	4.97±0.59	3.75±0.74
**Adiponectin (ng/mL)** ^‡^ ^a^	6147±312	4747±396*	4336±699	3611±136
**Leptin (pg/mL)** ^§^ ^a^ ^b^ ^c^	278.1±81.6	257.0±62.2	324.4±109.4	882.2±200.3*
**Free Fatty Acids (μM)** ^a^	118±1	148±2*	118±1	147±1*
**TC (mM)** ^b^	2.41±0.21	2.07±0.15	2.02±0.12	2.28±0.19
**HDL-C (mM)** ^b^	1.15±0.07	1.00±0.07	0.98±0.07	1.05±0.07
**LDL-C (mM)** ^b^	1.01±0.15	0.83±0.09	0.80±0.05	0.98±0.13
**TG (mM)**	0.56±0.03	0.55±0.04	0.54±0.04	0.56±0.07
**Pancreatic lipase (U/L)** ^a^	28±2	35±5	20±2	31±3
**Pancreatic Islet (μm** ^**2**^ **)** ^†^ ^b^	10.17 ± 1.08	11.49 ± 3.36	8.47 ± 2.36	5.58 ± 0.56

mean ±sem, n = 6,

^†^n = 6 to 9,

^‡^n = 4 to 6.

^a^Main effect of genotype at P<0.05.

^b^Main effect of diet at P<0.05.

^c^Interaction between genotype and diet at P<0.05 by linear mixed model analysis including 9-week-old mice on chow diet.

*P<0.05 *a priori* analysis by Wilcoxon rank sum test between WT and CD24KO within each diet

^§^detection threshold of 62.5 pg/mL.

**Fig 3:**
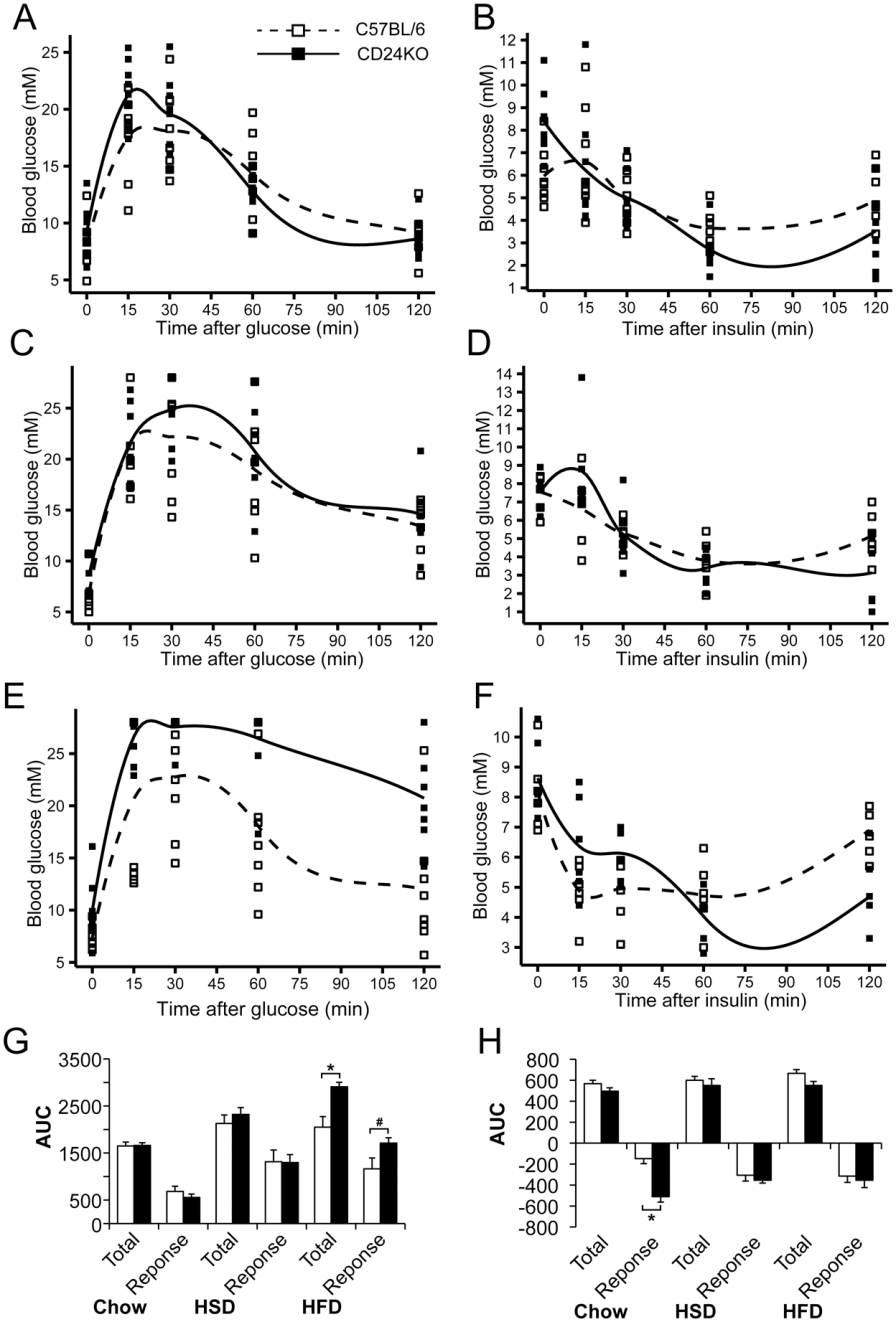
CD24KO male mice show altered glucose and insulin tolerance that is affected by adiposity-promoting diets. Male CD24KO and WT C57BL/6 mice were fed **A-B**. standard chow diet (chow), **C-D**. high sucrose diet (HSD), and **E-F.** high fat diet (HFD), as described in Fig 2. **A, C, E.** Blood glucose was determined after a 6 h fast and then 15 min, 30 min, 60 min, and 120 min following intraperitoneal injection of 2 mg/g glucose for glucose tolerance test. **B, E, F.** Blood glucose was determined after a 4 h fast and then 15 min, 30 min, 60 min, and 120 min following intraperitoneal injection of 1 mU/g bovine insulin for insulin tolerance test. Trend lines display the Loess conditional means and squares represent individual animals. The total glucose load (Total) and the glucose response from control levels (Response) for **G.** GTT and **H.** ITT were determined and compared between genotypes by Wilcoxon rank sum, *P<0.05, ^#^P = 0.09, n = 7–9 animals per group for glucose tolerance and 6–8 for insulin tolerance.

To determine the glucose and insulin responsiveness of CD24KO mice, we performed glucose tolerance tests (GTT) and insulin tolerance tests (ITT) in 9-week-old WT and CD24KO male mice (Fig 3). CD24KO and WT mice showed a statistically similar total glucose load and glucose response to GTT when on chow diet or HSD (Fig 3G). However, CD24KO mice on a HFD showed a significant increase in total glucose load and an increased glucose response approaching statistical significance (P = 0.09) (Fig 3G). Linear mixed model analysis revealed an interaction between diet and genotype that approached statistical significance (F[2, 42] = 2.6, P = 0.09). There was a significant effect of diet on GTT with respect to the glucose response (F[2, 42] = 16.0, P<0.001) with both HSD (P<0.001) and HFD (P<0.001) being significantly higher than chow. Therefore, adiposity-promoting diets affect the glucose sensitivity in both CD24KO and WT mice, with CD24KO displaying reduced glucose clearance when on HFD.

CD24KO mice fed chow diet showed a greater response to ITT than WT mice due to a delay in restoring blood glucose levels, while the overall glucose load remained similar (Fig 3H). When fed either HSD or HFD, CD24KO mice showed a statistically similar glucose response and total glucose load with ITT as WT mice (Fig 3H). Linear mixed model analysis revealed a significant interaction between genotype and diet in the glucose response to ITT (F[2, 34] = 5.3, P = 0.01) with a main effect of genotype (F[1,34] = 18.9, P = 0.0001) but no effect of diet (F[2,34] = 0.28, P = 0.76). Therefore, the apparent increased response of CD24KO to insulin is common to all diets, even though the difference is largest with chow diet.

### Reduced WAT depot mass in CD24KO mice is due to reduced adipocyte size

**Fig 4:**
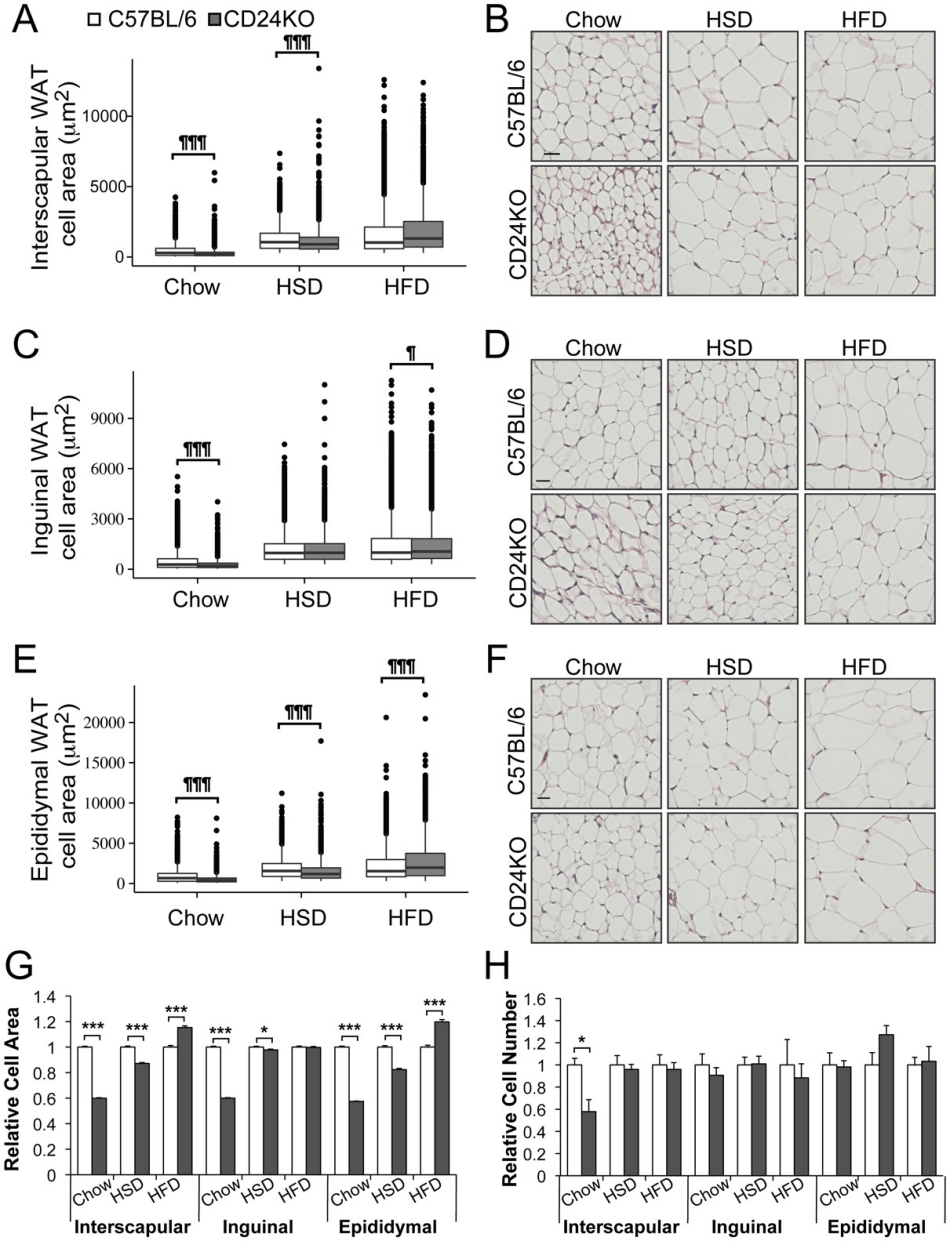
Adipocytes from CD24KO mice are hypotrophic. Male CD24KO and wild-type C57BL/6 mice were fed as described in Fig 2. At 9 weeks of age, **A-B.** interscapular WAT depots, **C-D.** inguinal WAT, and **E-F.** epididymal WAT were analyzed by H&E staining to determine adipocyte size. **A, C, E.** Data are shown as box-and-whisker plots with **B, D, F.** representative H&E images. Scale bar = 30 μm. Statistical differences in the distributions between WT and CD24KO were determined using the Kolmogorov-Smirnov test, ^¶^P<0.05, ^¶¶¶^P<0.001. **G.** Relative cell area compared to the WT on each diet from the respective WAT are shown. **H.** The relative cell number compared to the WT on each diet from the respective WAT is shown. Statistical significant was determined by Wilcoxon rank sum *P<0.05, ***P<0.001. n = 5–7, ≥225 cells were analyzed per animal.

WAT depots in the male CD24KO mice were analyzed for adipocyte cell size when fed chow diet or the adiposity promoting diets. We observed fewer large adipocytes and increased abundance of smaller cells in WAT from CD24KO compared to WT mice (Fig 4A–4G and S3 Fig). We observed no difference in brown fat adipocytes in WT compared to CD24KO mice (S2B Fig) The shift in size distribution of WAT adipocytes resulted in a significant reduction in the mean cell size in the CD24KO mice on chow diet in all depots (Fig 4G). When fed HSD, the mean cell size remained smaller in CD24KO in all depots however the relative difference in cell size was substantially reduced (Fig 4G). Interestingly, the inguinal depot no longer had a difference in the distribution of cell size in HSD-fed mice even though the mean cell size was modestly smaller (Fig 4C and 4G). When CD24KO were fed HFD, the distribution unexpectedly shifted to an increase in the frequency of larger cells and a decrease in the frequency of smaller cells compared to WT in all depots (Fig 4A, 4C, and 4E). The mean cell size of adipocytes in the interscapular and epididymal depots was significantly larger in the CD24KO than in WT mice (Fig 4G).

In order to determine if the number of cells was also affected by the lack of CD24, we estimated the number of adipocytes based on the mean weighted volumes, determined from the adipocyte cell area, and the weight of each WAT depot. There was no change in the number of adipocytes regardless of genotype, diet or depot, with the exception that CD24KO mice had fewer adipocytes in the interscapular depot when on chow diet (Fig 4H).

Overall, these data indicate that the reductions in fat pads of the CD24KO mice are due to hypotrophy but not hypoplasia when fed chow diet, and that hypertrophy is responsible for restoring WAT depot size when fed adiposity-promoting diets.

## Discussion

CD24 has previously been shown to be a marker of adipocytes progenitors *in vivo* [16,19] as well as a specific regulator of adipogenesis *in vitro* [18]. We have now established that the *in vivo* loss of CD24 in male mice leads to a generalized reduction of WAT, affecting both visceral and subcutaneous WAT as well as a number of metabolic disturbances.

The CD24-mediated reduction in WAT growth was sex-specific as male mice displayed a reduction in fat mass and percentage body fat while female mice did not show overt fat mass loss at any of the ages examined. Moreover, male CD24KO mice showed a significant increase in both lean mass and bone mass with no change in overall body mass. Therefore, in males, CD24 appears to promote the increase in fat and limit lean and bone mass during this rapid growth period. These observations are consistent with the divergence of mesenchymal stem cells to differentiate into osteoblast, myoblast, or adipocyte lineages where differentiation into one lineage actively inhibits differentiation into the other lineages [35]. Thus, these data suggest that CD24 expression may be necessary for optimal adipocyte differentiation, consistent with previous *in vitro* evidence [18], and that in the absence of CD24 osteoblast and myoblast differentiation is enhanced.

The protective factor or mechanism that prevents the CD24KO-mediated reduction of fat mass in female mice is not known. CD24 expression can be downregulated by activation of the estrogen receptor in human breast cancer cells [36]. Thus, it is reasonable to speculate that the increased circulation of estrogen may also downregulate CD24 expression in other tissues in female mice. If this is the case, then female mice may have developed compensatory mechanisms to allow for normal adipocyte development in the absence of CD24. The precise mechanism(s) for this sex-dependent rescue will require further investigation.

Based on the GTT and ITT data, CD24KO mice are capable of clearing glucose in response to an insulin challenge as well as mounting an insulin response to a glucose challenge. Visually, the CD24KO animals appear to have an increased total glucose load after GTT compared to WT mice on all three diets. However, this is due to the elevation of baseline fasting glucose in CD24KO mice. The response to glucose was only significantly increased when CD24KO were on HFD. This suggests that even though fasting insulin is lower in CD24KO than in WT, insulin is still being effectively mobilized to induce glucose clearance. Thus, the high plasma glucose and low insulin are not due to an inability to produce or respond to insulin on the chow diet. The GTT response and the initial response to the ITT demonstrate that glucose can be transported out of the blood in response to insulin with comparable kinetics to WT at the level of the whole organism. However, in response to ITT the blood glucose levels of CD24KO mice fell to below baseline levels during the timeframe when WT animals initiate recovery to baseline levels. Thus, the CD24KO mice appear to have a defect in re-mobilizing glucose to stabilize blood glucose levels after insulin challenge.

HOMA calculations revealed that 5-week-old CD24KO mice showed mild insulin resistance and decreased β-cell function that resolved by 9 weeks of age. Previous work has shown that CD24 is present in the developing pancreas and in developing exocrine cells surrounding the islets in adult tissue [37]. Therefore, this age-dependent shift in pancreatic islet function may represent a delay in pancreatic islet development. At 15 weeks of age, there was no difference in HOMA-IR but a decrease in β-cell function compared to WT, which may be due to impairment in the ability of the precursor cells to replace mature β-cells. This mild impairment is consistent with the observation that only 50% of all islets contain CD24-expressing cells [37]. However, we observed no significant effect of CD24KO on islet size, suggesting that any effect of CD24 in the pancreas is not due to major cellular or structural changes. These observations are consistent with the mild insulin resistance seen in 5-week-old mice by HOMA-IR and non-statistically significant increases in HOMA-IR with HSD and HFD. Furthermore, the elevation of fasting blood glucose and FFA at all ages and diets would be expected to induce insulin resistance in the CD24KO mice [38]. Therefore, future studies employing more sensitive measures of insulin resistance, such as insulin or glucose clamp studies [23] will be necessary to accurately determine the precise degree of insulin resistance in the male CD24KO mice on each diet.

The difference in the size of the insulin responsive compartments (i.e. fat vs. lean mass) between CD24KO and WT mice partially confounds the analysis of GTT and ITT with respect to adipocyte function since skeletal muscle is responsible for the majority of the glucose uptake [39]. Circulating glucose is taken up by adipocytes and skeletal muscle in response to insulin via the insulin-sensitive transporter GLUT4 [40]. Even though adipocytes only take up a small proportion of the total blood glucose, these cells regulate whole-body insulin sensitivity in part through GLUT4-mediated glucose uptake [41]. Therefore, an increase in the lean mass is expected to counteract insulin resistance induced by the increased adipocyte cell size seen in CD24KO on HFD. Even with the increased lean mass, HFD-fed CD24KO mice become less able to remove glucose from the circulation as evidenced by their increased response to GTT compared to their WT counterparts. Since hypertrophic adipocytes have reduced insulin-dependent glucose uptake up via GLUT4 that is associated with whole body insulin resistance [41,42], the increased GTT response in HFD-fed CD24KO may be due to dysfunctional adipocytes. However, the HFD-fed CD24KO remained responsive to ITT, suggesting that the response of muscle cells to insulin is maintained.

Insulin signaling promotes the transport of GLUT4 vesicles to the cell surface and simultaneously limits endocytosis of GLUT4 [43]. Comparatively, basal glucose uptake is mainly controlled by expression of GLUT1 via agents such as cAMP and endothelin-1 or stearoyl-CoA desaturase 1 [44,45]. We recently reported that CD24 regulates extracellular vesicle formation in B cells [46], which raises the possibility that CD24 may also regulate vesicle formation in pre-adipocytes to influence glucose transporter recruitment and/or recycling in pre-adipocytes to alter subsequent glucose uptake in mature adipocytes. Since fusion of GLUT4-containing vesicles is dependent on lipid raft-resident proteins [47–49], GLUT1 interacts with lipid raft proteins [50], and CD24 is able to regulate lipid raft occupancy [51,52]; regulation of lipid raft protein localization is one potential mechanism that may link CD24 to glucose uptake. Additional study will be required to identify whether a connection exists between CD24, glucose uptake, and the observed metabolic dysfunction, as well as the potential contribution of skeletal muscle to the regulation of glucose metabolism in the CD24KO mice.

CD24KO mice demonstrate an increase in both circulating glucose and FFA levels with all diets. Circulating levels of glucose and FFA can be influenced, at least partially, by a deficiency of lipogenesis in WAT or increased lipolysis of WAT, to synthesize or degrade TG, respectively. However, we did not observe a decrease in the mRNA expression of 4 select genes involved in regulating adipogenesis (*PPAR-γ*), lipogenesis (*Fasn* [53]), and lypolysis (*Atlgl* and *Hsl*) [54] (S4A Fig). Therefore, the absence of CD24 does not substantially interfere with expression of these key lipid-regulating genes in adipocytes. The regulation of lipogenesis and lipolysis depends on both the expression level and the protein activity of these and many other genes to regulate multiple processes during adipogenesis such as fatty acid synthesis (e.g. ACL, ACC, SCD), glucose transport (e.g. GLUT4), fatty acid transport (e.g. LPL, FABP2), TG synthesis (e.g, AGAT, MGAT, DGAT), and lipid droplet maintenance (e.g. Plin1) [53,54]. Alternatively, the increase in FFA levels may be due to deficiencies in fatty acid oxidation in the liver or skeletal muscle. Adiponectin stimulates fatty acid oxidation and glucose uptake in the liver and skeletal muscle [55]. Therefore, the increased levels of glucose and FFA may be due to the decreased levels of circulating adiponectin in the CD24KO mice in response to the reduction in overall WAT mass.

CD24 has been shown to be an important marker of pre-adipocytes [16,18,19]. We determined if the reduction in fat mass that we observed in the CD24KO mice was due to a reduction in the number of pre-adipocytes (hypoplasia) or due to a reduction in adipocyte size (hypotrophy). Adipocytes from CD24KO mice were approximately half the size of those from WT mice in both subcutaneous and visceral depots. In contrast, we saw no change in the estimated cell number in any depot from CD24KO mice on any diet, with the exception of a reduction in cell number in the interscapular WAT depot from chow-fed mice. Of note, there was no obvious WAT necrosis observed in the histological sections or in the gross fat depots. Finally, we did not observe any significant differences in the percentage of CD34^+^CD31^-/lo^CD140a^+^ pre-adipocytes from adherent SVF cultures from CD24KO mice compared to WT mice (S4B Fig). Therefore, we can conclude that the main cause of the reduction in fat pad mass is due to a deficiency in the development of small adipocytes to large adipocytes and not to a significant reduction in the generation of pre-adipocytes or loss of mature adipocytes.

Previous experiments in 3T3-L1 pre-adipocytes showed that prevention of the cAMP/glucocorticoid-mediated increase in CD24 significantly decreased the subsequent upregulation of genes responsible for adipocyte differentiation and the associated lipid accumulation [18]. The discrepancy in the effect of CD24 on adipocytes *in vitro* (a reduction in overall adipogenesis [18]) and *in vivo* (a reduction in mature adipocyte size) data is likely due to a combination of factors. Firstly, *in vivo* there is a continual renewal of the pre-adipocyte population from mesenchymal stem cells and a reduction in ability of pre-adipocytes to fully differentiate could be compensated by an increase in the number or rate of pre-adipocytes entering the differentiation process. This is precisely what occurs during B lymphocyte development in CD24KO mice [21]. With respect to lipid accumulation *in vitro*, we analyzed the entire population of cells including those that did not differentiate [18], while the *in vivo* analysis of cell size is strictly on mature adipocytes that have differentiated successfully. As we did not analyze the lipid accumulation of the 3T3-L1 cells with reduced CD24 expression on a per cell basis, it is not known if lipid droplet size was affected *in vitro*. Lastly, if CD24 is facilitating glucose uptake in an adipose-specific manner, deficiencies in lipid formation would be more apparent in 3T3-L1 cells as these are heavily dependent on glucose for the generation of glycerol-3-phosphate (G3P) necessary for *de novo* fat synthesis [41,45] whereas glyceroneogenesis can play an important role in G3P generation in adipose tissue *in vivo*, particularly if intracellular glucose is limiting [56,57]. Thus, *in vivo* CD24KO pre-adipocytes can take advantage of the non-carbohydrate G3P precursors lactate, pyruvate, alanine, glutamine, and glutamate [58], whereas only glutamine is available *in vitro* [18]. The increased availability of these precursors that occurs in response to adiposity-promoting diets [56,59,60] along with the increased circulating TG could further promote lipid droplet expansion.

The adiposity promoting diets were able to rescue the WAT mass in CD24KO mice. Therefore, the absence of CD24 did not impede the ability of adipocytes to incorporate the increased circulating TG, either from VLDL or chylomicrons [32,33]. In fact, in relative terms, the CD24KO mice showed an increased response, considering that the fat pad weights in CD24KO mice fed chow diet were significantly smaller than WT mice but became similar to WT when CD24KO were fed HSD or HFD. Therefore, CD24 does not regulate adipocyte cell size directly but may play a role in regulating nutrient or energy sensing. Previous *in vivo* analyses of CD24 expression suggested that CD24 is important for the commitment of adipocyte progenitors to committed adipocyte precursors [19], and our *in vitro* work showed that CD24 expression peaks early during adipogenesis [18]. Together, these data suggest that CD24 likely functions prior to the development of mature adipocytes. Consistent with this and other work showing that CD24 expression responds very rapidly to diet [20], as well as our observation that fasting glucose and FFA levels were increased in CD24KO mice on all diets, an alternative possibility for CD24 function is that CD24 regulates the ability of pre-adipocytes to sense extracellular glucose or FFA levels. This change in energy-sensing abilities may then increase the threshold for inducing TG synthesis, thus reducing lipid droplet expansion in the mature adipocyte. Therefore, although both the *in vivo* and *in vitro* data support a role for CD24 in regulating some aspect of glucose sensing, uptake, or metabolism, future studies are necessary to determine the precise mechanism used by CD24 to regulate adipocyte cell growth.

Overall, the clear reduction in WAT mass and size in all three depots examined with no effect on BAT or overall body size supports a role for CD24 in regulating WAT growth and development. However, some of the observed differences may be influenced by minor lineage differences, in addition to the absence of CD24, since the CD24KO mice were not directly compared to WT littermates due to the very low transmission of homozygous knock-out offspring from heterozygous matings [21]. Similarly, C57BL/6 mice are known to vary in their response to diet-induced obesity [61], which in combination with the absence of CD24 increases the variability of the responses. Nevertheless, the statistical power of our analyses was at 0.82 or greater at an alpha of P<0.05 for all of the metabolic parameters found to be significantly different, even within the smallest sample sizes collected from 15-week WT mice. Thus, we are confident of the differences found between CD24KO and WT but acknowledge that larger studies would be needed to address the subtle metabolic disturbances in the measures that were not found to be statistically significant at 15 weeks of age. In addition, since these mice were analyzed primarily at 9 weeks of age, when percentage body fat was reduced but overall body mass was unaffected, analysis of older mice may reveal a different degree of insulin or glucose tolerance, and/or response to adiposity-promoting diets.

Understanding the regulation of fat storage has depended largely on the study of mouse models of lipodystrophy, many of which have been generated based on recapitulating the mutations identified in human lipodystrophic syndromes. These include mice with mutations in *AGAT2*, *BSCL2*, and *CAV1*, that show profound losses in WAT and effects on lipid and glucose metabolism [2]. An analysis of 13 murine models of lipodystrophy identified the reduction in fat pad mass, elevated TG, insulin resistance, elevated blood glucose, liver steatosis, and the decreased production of leptin and adiponectin as some of the features of the various lipodystrophic mouse models [62]. However, no one model displays all of the phenotypes associated with human lipodystrophic syndromes [15]. For example, out of the 13 models, only six develop liver steatosis, ten are insulin resistant, and six display a dysregulation of glucose. In contrast, the reduction in fat mass, along with a reduction of leptin and adiponectin, were common to all of the models analyzed. Similarly, CD24KO mice display only a minority of the physiological and metabolic abnormalities associated with human lipodystrophic syndromes [15]. Notably, unlike the mouse models and human syndromes, the WAT from CD24KO mice is responsive to adiposity-promoting diets. Nevertheless, the availability of the CD24KO mouse as another model to study alterations in TG storage and metabolism will increase our understanding of this essential organ.

CD24 has not been associated with impaired fat accumulation in humans; however, given the moderate loss of WAT that is restored by adiposity-promoting diets it is unlikely that this connection would be observed clinically. Polymorphisms in CD24 have been associated with risk of immune-associated diseases such as multiple sclerosis [63], systemic lupus erythematosus [64], and irritable bowel syndrome [65]. However, the relationship of these diseases of overactive immunity to obesity or lipodystrophy is unclear. Therefore, future targeted work is necessary to determine the relevance of CD24 to human disorders of WAT.

In summary, this study correlates with previous *in vitro* work showing that CD24 is a modulator but not a master regulator of fat cell development [18]. The clear reduction in WAT mass and size in all three depots examined with no effect on BAT or overall body size supports a role for CD24 in regulating WAT growth due to either cell autonomous or inter-organ effects. These data further point to the involvement of CD24 in maintaining glucose and free fatty acid homeostasis that is independent of diet.

## Supporting Information

S1 Fig CD24KO female mice have no significant fat reduction but increased lean and bone weight compared to wild-type C57BL/6 male mice. **A.** Total fat weight, **B.** percent fat weight, **C.** total lean weight, **D.** percent lean weight, **E.** bone weight, **F.** percent bone weight, and **G.** estimated total body weight from dual X-ray absorptiometry (DEXA) scans of female wild-type C57BL/6 and CD24KO mice at 5, 9, and 12 weeks of age on standard show diet. Trend lines display the Loess conditional means and squares represent individual animals. Scans and calculations exclude the head. Statistical significance was determined by repeated measures ANOVA, n = 6, interaction effects between genotype (G) and time (T) are indicated as P(G*T), main effects of genotope are shown as P(G) and the effect of time within each genotype is shown as P(G[T]).
(TIF)

S2 Fig Morphology of BAT and liver does not differ in CD24KO and WT mice. Representative images of **A.** whole BAT (scale bar = 1 cm) and **B.** H&E stained BAT (scale bar = 30 μm) from 9-week-old C57BL/6 and CD24KO mice fed standard chow diet (chow), high sucrose diet (HSD), or high fat diet (HFD), n = 3–7. **C.** Representative images from liver sections stained with Oil Red O from 9-week-old C57BL/6 and CD24KO mice fed standard chow diet (chow), high sucrose diet (HSD), or high fat diet (HFD). Scale bar = 50 μm). **D.** The average percentage of the liver stained with Oil Red O was calculated and is shown as box-and-whisker plots. Statistical significance was determined by 2-way ANOVA followed by Tukey HSD post-hoc analysis. *P<0.05, **P<0.01, n = 7–11. There was no significant effect of genotype.
(TIF)

S3 Fig Distribution of adipocyte cell size. Male C57BL/6 and CD24KO mice were fed as described in Fig 2. Adipocyte size from **A.** interscapular, **B.** inguinal, **C.** epididymal WAT depots from mice fed standard chow diet (chow), high sucrose diet (HSD), or high fat diet (HFD), as indicated, were analyzed for adipocyte cell size using 200 μm^2^ bins. Frequency histograms of data from Fig 4 are shown, n = 5–7, ≥225 cells were analyzed per animal.
(TIF)

S4 Fig There is no significant change in WAT gene expression of *PPARγ*, *Fasn*, *Atgl*, or *Hsl* or in the percentage of CD34^+^CD31^-/lo^CD140a^+^ pre-adipocytes in CD24KO mice. **A.** Total RNA was isolated from epididymal WAT depots from 9-week-old male CD24KO and WT C57BL/6 mice fed chow diet. Relative gene expression of fatty acid synthase (*Fasn*), peroxisome proliferator-activated receptor γ (*Pparg)*, Adipose triglyceride lipase (*Atgl*), and hormone sensitive lipase (*Hsl*) normalized to *RPL-P0*, was determined. Data are shown as mean ± sem of the Log2 expression level relative to WT. Statistically significant differences of each gene between WT and CD24KO was analysed by unpaired Student’s T-test and found to be not significant, n = 5. **B.** Percentage of cells staining CD34^+^CD31^-^CD140a^+^ from isolated SVF after overnight culture is shown for 5-week-old male CD24KO and WT C57BL/6 mice fed chow diet. CD34 and CD31 are markers of mesenchymal stem cells and endothelial cells, respectively, and CD140a (PDGFR-α) is a marker for pre-adipocytes *in vivo*. Statistically significant differences between WT and CD24KO were analysed by unpaired Student’s T-test and found to be not significant, n = 3.
(TIF)
