# Supplementary figures and images for: Loss of CD24 in Mice Leads to Metabolic Dysfunctions and a Reduction in White Adipocyte Tissue
Source: PLoS One. 2015 Nov 4;10(11):e0141966. doi: 10.1371/journal.pone.0141966 (PMC4633231; doi:10.1371/journal.pone.0141966)

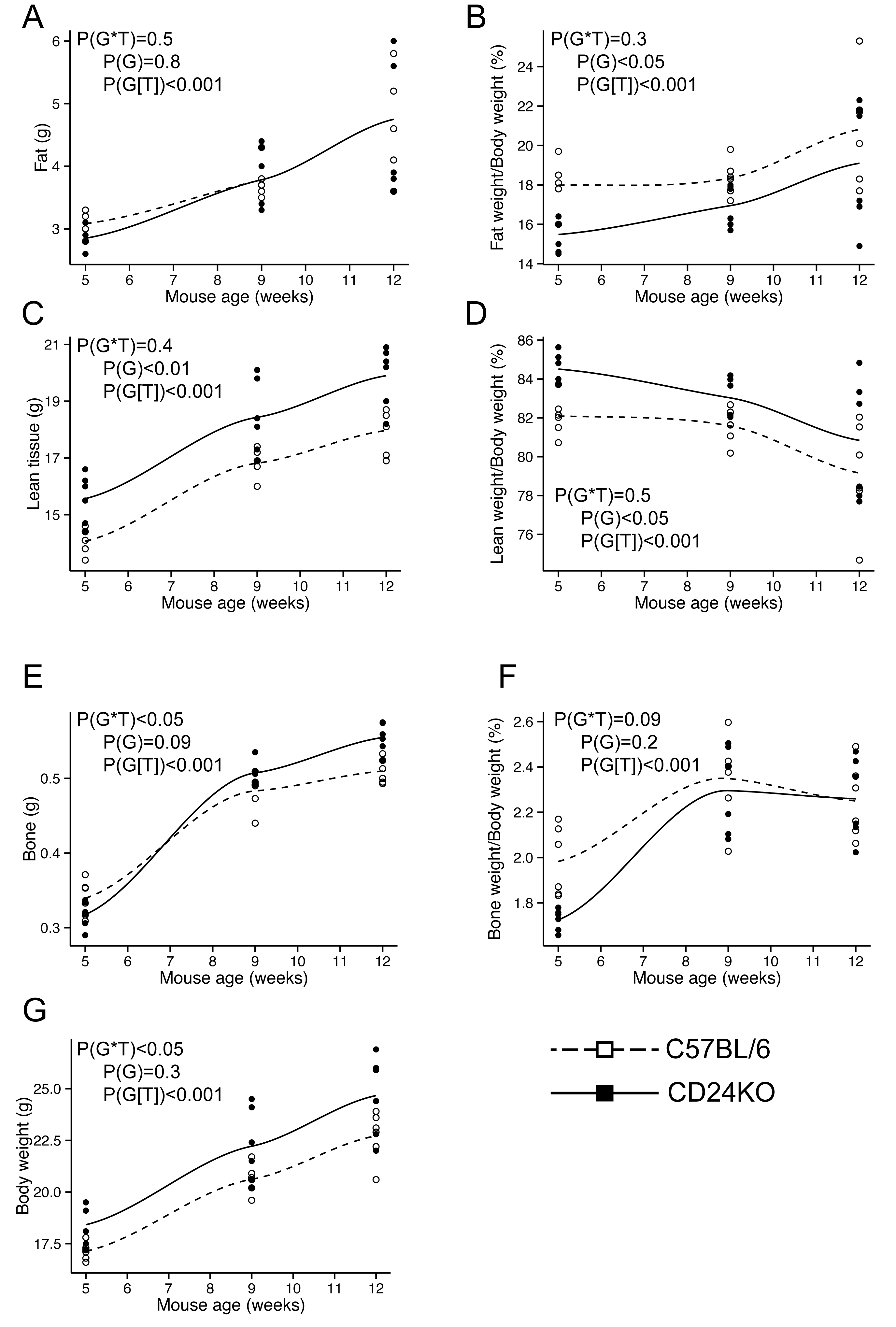

Supplement: S1 Fig — A. Total fat weight, B. percent fat weight, C. total lean weight, D. percent lean weight, E. bone weight, F. percent bone weight, and G. estimated total body weight from dual X-ray absorptiometry (DEXA) scans of female wild-type C57BL/6 and CD24KO mice at 5, 9, and 12 weeks of age on standard show diet. Trend lines display the Loess conditional means and squares represent individual animals. Scans and calculations exclude the head. Statistical significance was determined by repeated measures ANOVA, n = 6, interaction effects between genotype (G) and time (T) are indicated as P(G*T), main effects of genotope are shown as P(G) and the effect of time within each genotype is shown as P(G[T]). (TIF) [file pone.0141966.s001.tif]

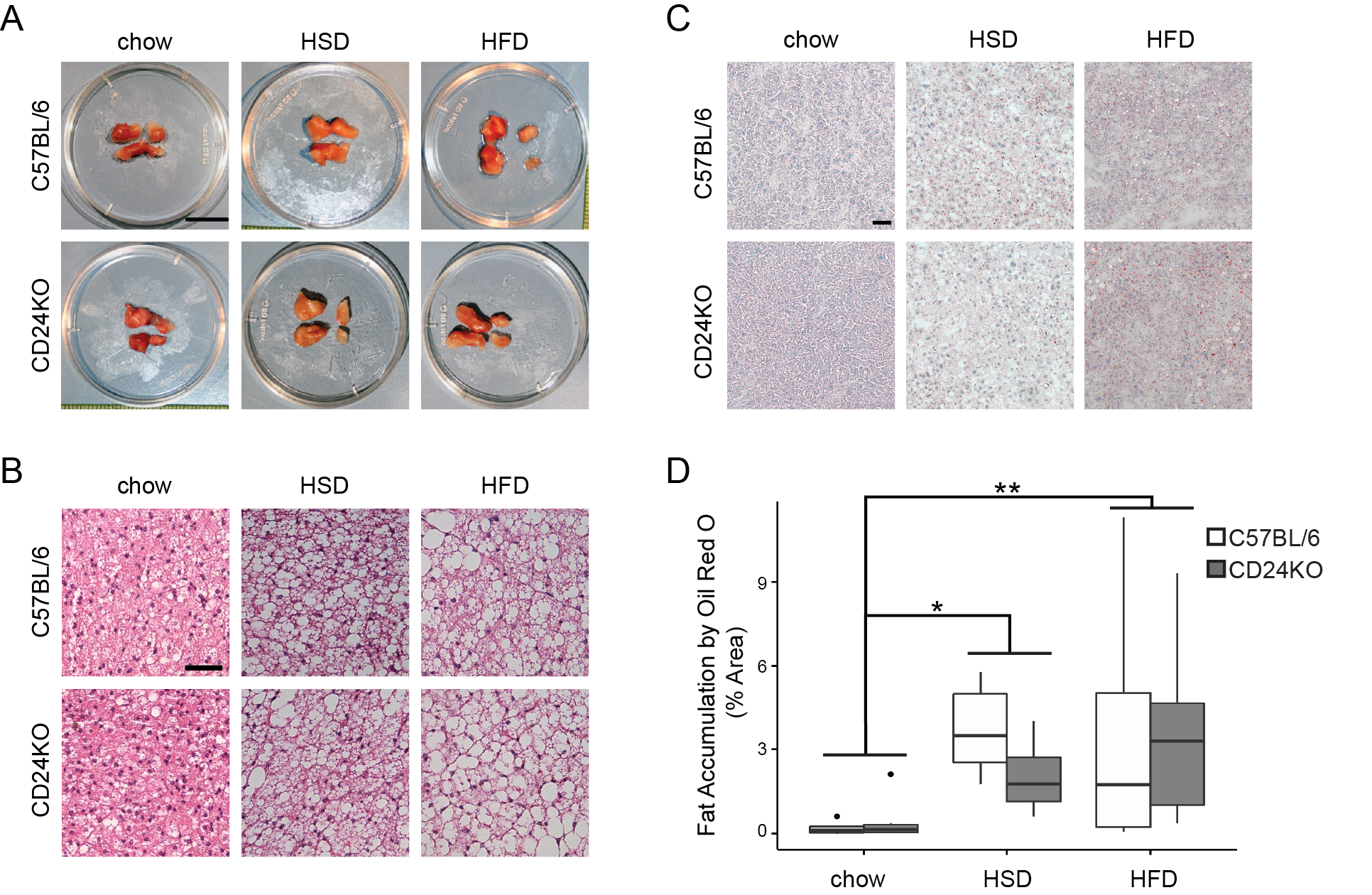

Supplement: S2 Fig — Representative images of A. whole BAT (scale bar = 1 cm) and B. H&E stained BAT (scale bar = 30 μm) from 9-week-old C57BL/6 and CD24KO mice fed standard chow diet (chow), high sucrose diet (HSD), or high fat diet (HFD), n = 3–7. C. Representative images from liver sections stained with Oil Red O from 9-week-old C57BL/6 and CD24KO mice fed standard chow diet (chow), high sucrose diet (HSD), or high fat diet (HFD). Scale bar = 50 μm). D. The average percentage of the liver stained with Oil Red O was calculated and is shown as box-and-whisker plots. Statistical significance was determined by 2-way ANOVA followed by Tukey HSD post-hoc analysis. *P<0.05, **P<0.01, n = 7–11. There was no significant effect of genotype. (TIF) [file pone.0141966.s002.tif]

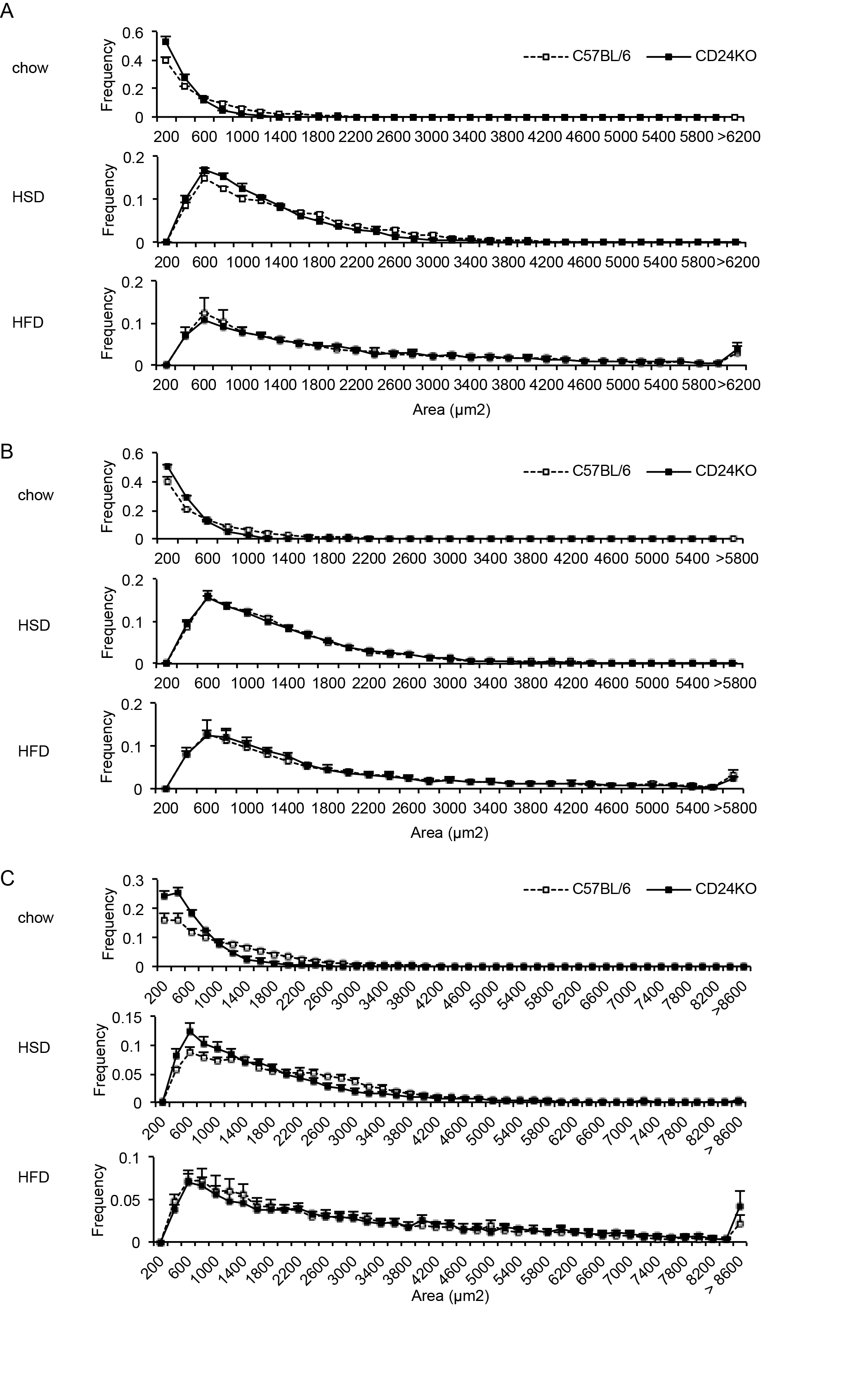

Supplement: S3 Fig — Male C57BL/6 and CD24KO mice were fed as described in Fig 2. Adipocyte size from A. interscapular, B. inguinal, C. epididymal WAT depots from mice fed standard chow diet (chow), high sucrose diet (HSD), or high fat diet (HFD), as indicated, were analyzed for adipocyte cell size using 200 μm2 bins. Frequency histograms of data from Fig 4 are shown, n = 5–7, ≥225 cells were analyzed per animal. (TIF) [file pone.0141966.s003.tif]

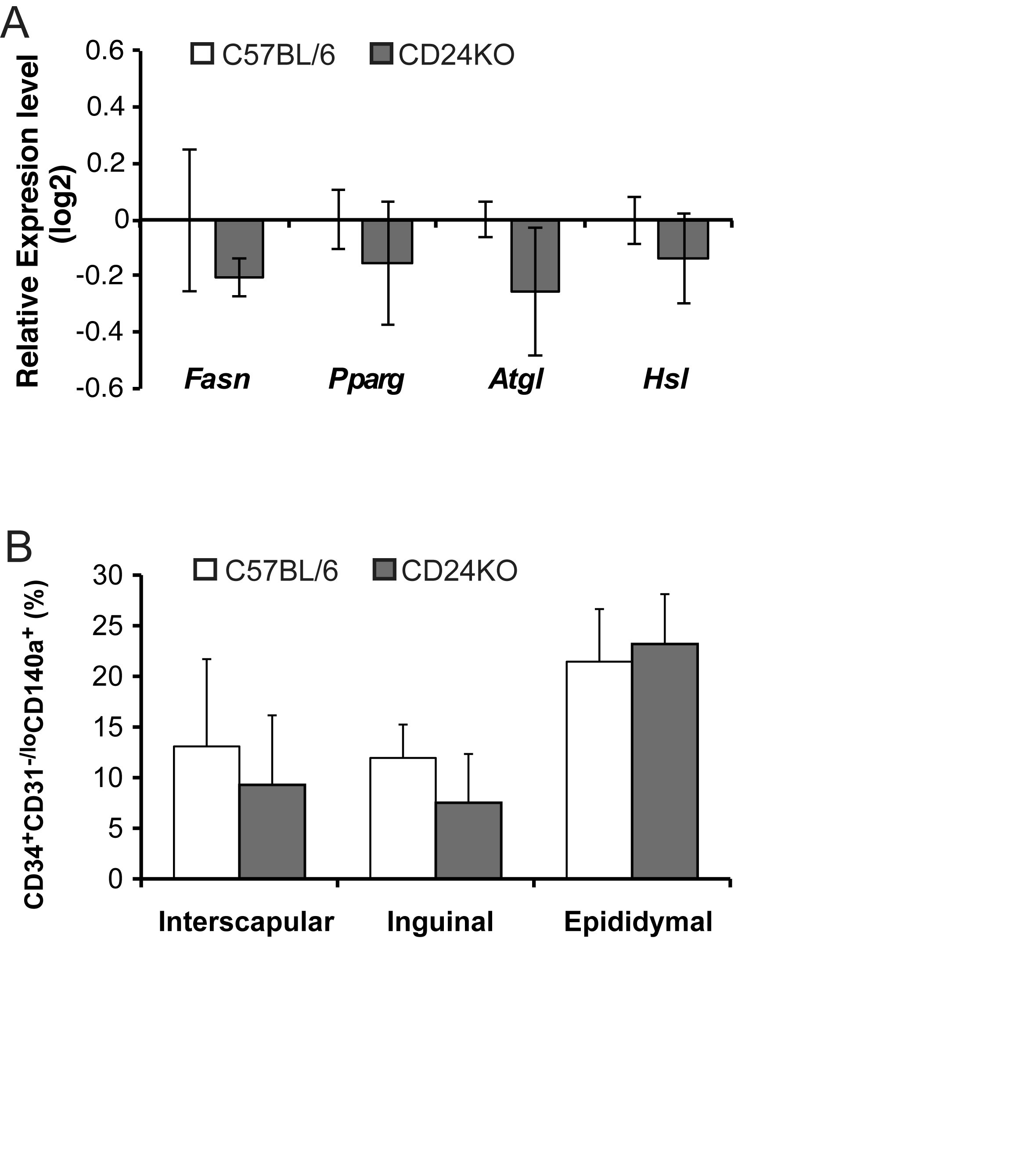

Supplement: S4 Fig — A. Total RNA was isolated from epididymal WAT depots from 9-week-old male CD24KO and WT C57BL/6 mice fed chow diet. Relative gene expression of fatty acid synthase (Fasn), peroxisome proliferator-activated receptor γ (Pparg), Adipose triglyceride lipase (Atgl), and hormone sensitive lipase (Hsl) normalized to RPL-P0, was determined. Data are shown as mean ± sem of the Log2 expression level relative to WT. Statistically significant differences of each gene between WT and CD24KO was analysed by unpaired Student’s T-test and found to be not significant, n = 5. B. Percentage of cells staining CD34+CD31-CD140a+ from isolated SVF after overnight culture is shown for 5-week-old male CD24KO and WT C57BL/6 mice fed chow diet. CD34 and CD31 are markers of mesenchymal stem cells and endothelial cells, respectively, and CD140a (PDGFR-α) is a marker for pre-adipocytes in vivo. Statistically significant differences between WT and CD24KO were analysed by unpaired Student’s T-test and found to be not significant, n = 3. (TIF) [file pone.0141966.s004.tif]
